# DensityMap: a genome viewer for illustrating the densities of features

**DOI:** 10.1186/s12859-016-1055-0

**Published:** 2016-05-06

**Authors:** Sébastien Guizard, Benoît Piégu, Yves Bigot

**Affiliations:** UMR INRA-CNRS 7247, PRC, Centre INRA Val de Loire, 37380 Nouzilly, France

**Keywords:** Genome, Visualization, Annotation, GFF

## Abstract

**Background:**

Several tools are available for visualizing genomic data. Some, such as Gbrowse and Jbrowse, are very efficient for small genomic regions, but they are not suitable for entire genomes. Others, like Phenogram and CViT, can be used to visualise whole genomes, but are not designed to display very dense genomic features (eg: interspersed repeats). We have therefore developed DensityMap, a lightweight Perl program that can display the densities of several features (genes, ncRNA, cpg, etc.) along chromosomes on the scale of the whole genome. A critical advantage of DensityMap is that it uses GFF annotation files directly to compute the densities of features without needing additional information from the user. The resulting picture is readily configurable, and the colour scales used can be customized for a best fit to the data plotted.

**Results:**

DensityMap runs on Linux architecture with few requirements so that users can easily and quickly visualize the distributions and densities of genomic features for an entire genome. The input is GFF3-formated data representing chromosomes (linkage groups or pseudomolecules) and sets of features which are used to calculate representations in density maps. In practise, DensityMap uses a tilling window to compute the density of one or more features and the number of bases covered by these features along chromosomes. The densities are represented by colour scales that can be customized to highlight critical points. DensityMap can compare the distributions of features; it calculates several chromosomal density maps in a single image, each of which describes a different genomic feature. It can also use the genome nucleotide sequence to compute and plot a density map of the GC content along chromosomes.

**Conclusions:**

DensityMap is a compact, easily-used tool for displaying the distribution and density of all types of genomic features within a genome. It is flexible enough to visualize the densities of several types of features in a single representation. The images produced are readily configurable and their SVG format ensures that they can be edited.

**Electronic supplementary material:**

The online version of this article (doi:10.1186/s12859-016-1055-0) contains supplementary material, which is available to authorized users.

## Background

Visualizing the ever-increasing amounts of DNA sequence data for genomic purposes is becoming a great challenge [1]. One solution is to develop genome browsers. The first, and probably the most popular, was the UCSC Genome Browser, which was released in 2002 and used to display human genomic data [2]. Several others, including Gbrowse, JBrowse, Abrowse and Annot-J [3], are now available. They are ergonomically more efficient than the original and include new functions, such as collaborative annotation with web Appollo [4]. These browsers are useful for displaying discrete chromosome regions but are not suitable for visualizing whole chromosomes.

Other tools have been developed for visualizing whole chromosomes. One of the most widely used is Circos [5, 6], which represents chromosomes by arranging them on a circle. It can also be used to plot annotations, quantitative data and relationships between parts of different chromosomes or genomes [7]. However, Circos representations become dense as their complexity increases, which alters the efficacy of their visualization. Two new programs designed to simplify visualization of whole chromosome sequences were released recently. PhenoGram [8] represents chromosomes and uses ideograms, lines, and different coloured symbols to locate information like phenotypes, genes, CNVs, SNPs, etc. While the PhenoGram web-interface is user-friendly, it requires the input files to be in a specific tabulated format rather than a standard format like Generic feature format (GFF), the most common format for annotation files. It also cannot display the density of a specific feature at a given position in a chromosome. CviT (ChromosomeVisualization Tool) [9] circumvents these limitations. It can represent chromosome contents from a GFF file, is readily configurable and the output image can be customized. CViT can also plot the densities of some features along chromosomes using histograms placed beside the chromosome representation. This tool produces reliable images when the features are not too dense but becomes limited when the density of a feature like interspersed repeats or DNA motifs is high. CViT must also use a GFF file that contains the density of a feature for a given set of windows along a chromosome. As Cvit is not designed to compute these densities, the GFF file must be revised each time the window width is changed. We have therefore developed a program, DensityMap.pl, inspired by CviT, which can produce maps that include the densities of one or more types of features while displaying the whole genome in a chromosome.

## Implementation

DensityMap is run with Perl script in the command line and uses the GD::SVG Perl package to produce SVG pictures. DensityMap computes a representation of the density of a feature on chromosomes using one GFF file (GFF2, GFF2.5 or GFF3) describing a chromosome as input. The program plots as many density maps along a chromosome as there are features specified. It can plot a density map for the plus strand, minus strand, or the plus and minus strands, combinations of plus and minus strands, or plus, minus and compiled strands, for each feature. Density is computed using a tilling window without overlap whose length is fixed by the user or automatically computed to produce an output image that fits the maximum image size. All this information can be set by the user in the command line. DensityMap also automatically calculates the density of a feature for each pixelized region of a chromosome, whatever the representation scale used. The way the density of a feature varies along a chromosome is represented using a colour scale from 0 to 100 %. A single colour scale can be used for all features investigated or each feature can have is own colour scale. Like CViT, DensityMap.pl produces visualizations that are fully configurable in a Scalable Vector Graphics (SVG) format. This makes it easy to edit high quality images for publication. The program also includes graphical options for configuring almost all elements (margins, map width, scale, etc.) of the image. The options are shown in Table 1.

**Table 1:** DensityMap options

Short	Long	Type	Description
Mandatory options			
-i	--input	string	GFF file name
-re	--region_file	string	A BED file describing sequence regions to plot. It allow to plot specific regions and not the whole seq. Example of file content: 2L[TAB]100000[TAB]200000 2R[TAB]300000[TAB]450000
-o	--output_img_name	string	output image name
-ty	--type_to_draw	string	Type (column 3 of GFF) to draw, strand(s) to plot and colour scale to use **Type:** Match, gene, CDS, etc. **Strand:** - - > strand – (1 Density Map (or DM)) + − > strand + (1 DM) both - > strand - and strand + (2 DM) fused - > Combination of strand - and strand + (1 DM) all - > strand - and strand + and fused (3 DM) **Format:** “Type1 = Strand = colour_scale” **i.e.:** “match = all = 7;gene = both = 4;CDS = fused = 10”
Generic options			
-for	--force	none	Automatically answers yes to picture size validation
-v	--verbose	none	Activate verbose
-h	--help	none	Print help
Density options			
-c	--colour_scale	integer	Number of the colour scale to use
-sc	--scale_factor	integer	Window length (in base pairs) to use
-a	--auto_scale_factor	integer	Maximum picture height in pixels
-ro	--rounding_method	string	Rounding densities with floor or ceiling
-gc	--gc	integer	Colour scale number for density map of the GC % of chromosome, Requires the presence of the sequence in ##FASTA section of the GFF file
Graphical options			
-ti	--title	string	Picture title
-w	--win_size	integer	Picture height in pixels
-sh	--show_scale	integer	Draw scale, with the integer indicating the maximum number of ticks to print on the scale
-str_w	--strand_width	integer	Strand width in pixels
-str_s	--strand_space	integer	Space between strands in pixels
-sp	--space_chr	Integer	Space between chromosomes
-lm	--lmargin	integer	Left margin in pixels
-rm	--rmargin	integer	Right margin in pixels
-tm	--tmargin	integer	Top margin in pixels
-bm	--bmargin	integer	Bottom margin in pixels
-ba	--background	integer	Picture background colour
-la	--label_strand_rotation	integer	Rotation (in degrees) of strand label
-ft_f	--ft_family	string	Text font
-ft_s	--ft_size	integer	Font size

The program computes the size of the output image according to the number of chromosomes (GFF files), the number of features to represent, the number of strands to plot and the window size. If the user chooses automatic scale computing, the program calculates a windows size that gives an image that lies within the maximum image size defined by the user. The program asks the user to check the output picture size before processing the data. It then builds the image by adding the various graphical elements (background, title, scale) and processes the data for plotting the chromosome strands. It sequentially opens GFF files, filter features (GFF file third column) selected by the user with the option -ty (types). The intervals are collected and sorted by their beginnings and merged to remove overlaps. Lastly, the program computes the densities - (number of bases covered by the feature /window size) x 100 - and then draws it within the image. A synopsis of the main algorithm and functions is supplied in Additional file 1 and a manual in Additional file 2.

Even if the main purpose of DensityMap is to plot whole genome data, it can be interesting to compare specific loci of several sequences. This can be done using the --region_file option. The user has to provide a BED file - a tabular formatted file compound of three column where the first column design the sequence, the second the region start position and the third region end position - describing the region of interest on each sequence. In addition to the density map, the program produce a CSV file - a tabular formatted file - that contain the densities computed for all features, windows and sequences.

## Results

**Fig. 1:**
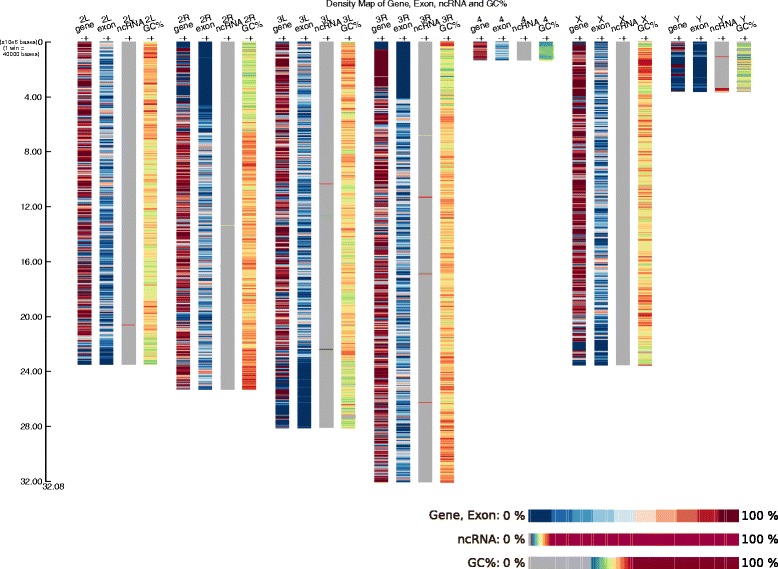
Density map of genes, exons, ncRNA and GC % in chromosomes of *D. melanogater*. The command line was: DensityMap.pl -i dmel.gff3 -o egn -ty ‘gene = fused;exon = fused;ncRNA = fused = 10’ -gc 12 -sc 40000 -ba white -str_s 15 -str_w 25 -sp 35 -sh 50 -title “Density Map of Gene, Exon, ncRNA and GC%” -la −15 -ro ceil. The arms of chromosomes 2 and 3 are split into two annotation files 2 L, 2R and 3 L, 3R. Four density map were drawn for each chromosome, one each for genes, exons, ncRNA and GC%. Tilling windows were 40,000 bp long. Densities are represented with three colour scales. That for genes and exons is blue - red with colour tone changes for each 10 % density change. The second, for ncRNA, shows 0 % density as grey, densities of 1 to 9 % are represented by a blue—red colour gradient, and densities of 10 %l or greater by dark red. The third colour scale, for GC content, shows densities below 30 % in grey, 30–49 % as a green—red colour gradient, and densities of 50 % and above in dark red. The scale is in Mbp

We have used DensitMap to examine two examples based on data on the genome of *Drosophila melanogaster (*available at http://flybase.org). The first (Fig. 1) illustrates the capacity of DensityMap to represent features that occur very frequently in a genome. This study is of the genes, exons, regions coding ncRNAs and the GC content of *D. melanogaster* chromosomes. The image produced shows that genes cover very large regions of the chromosomes, are absent from the centromeres and less frequent on the Y chromosome. As expected, the distribution of exons agrees with that of the genes. The representation of the GC content shows that the centromeres are GC-poor while the regions covered by genes are GC-enriched. The terminal regions are different of the rest of the X chromosome in that they are very GC-rich. The image also shows that ncRNAs are evenly distributed throughout the chromosomes, except for the centromeres and chromosome Y and a few regions where the ncRNA density is over 10 %.

**Fig. 2:**
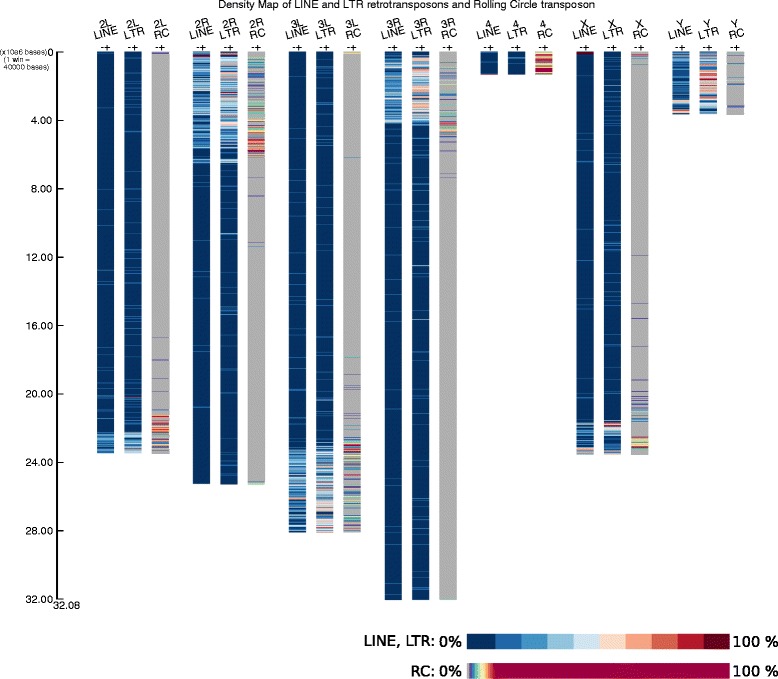
Density map of LINE and LTR retrotransposons and rolling-circle transposons (RC) in *D. melanogaster*. The command line was: DensityMap.pl -i dmel.gff3 -o te -ty 'LINE = fused;LTR = fused;RC = fused = 10′ -sc 40000 -ba white -str_s 15 -str_w 25 -sp 35 -sh 50 -title “Density Map of Gene, Exon, ncRNA and GC%” -la −15 -ro ceil. The arms of chromosomes 2 and 3 are shown in two annotation files 2 L, 2R and 3 L, 3R. Two density maps were drawn for each chromosome, one for LINE retrotransposon and one for LTR transposon. Tilling windows were 40, 000 bp long. The densities of LTR or LINE are shown as a blue- red gradient with 10 % intervals. Zero % RC is shown in grey. Densities of 1 to 9 % are shown in dark blue to red, and those over 10 % are in dark red. The scale is in Mbp

The second example illustrates the ability of DensityMap to produce images describing features that occur at extreme (high or low) densities. We looked at the distributions and densities of three kinds of transposable elements (TEs): LTR and LINE retrotransposons and rolling-circle transposons. Rolling-circle transposons like helitrons are present in this genome, but they are much less abundant than LTR or LINE retrotransposons. These features were visualized with colour scales that were appropriate for features present at low density (Fig. 2). The default program setting rounds down values using a floor method that transforms values between 0 and 1 to 0. But, in this case, we selected the ceiling method, which rounds up values between 0 and 1 to 1 and are thus visualized. The densities of the LTR and LINE retrotransposons can also be visualized. Their distributions in the *D. melanogaster* genome are similar, except that LTRs are very dense in the inner regions of the Y chromosome while most LINEs are present at one end. The TEs in chromosomes 2 and 3 are clustered in the telomeres. A large intra-chromosomal region is devoid of repeated elements. Rolling circle transposons are concentrated at the ends of chromosomes 2 and 3 and the arms of the Y chromosome. The red windows seem to indicate helitron hotspots. Helitrons are also present in the inner regions of chromosomes but their densities are very low. There are two hotspots of these TEs on the X chromosome, one in each telomere; they are absent from most of the other regions. The density of helitrons in most regions of chromosome 4 is over 10 %.

## Conclusion

The development of sequencing technologies has led to improvements in genome sequence models—they have become better adapted and much more varied. This, in turn, has led to the development of tools for analysing the genome models, such as genome browsers. While these tools are most useful for viewing small regions of chromosomes, very few provide an overall view of the complete genome. CViT and Phenogram provide two solutions, but they also have limitations: non-standard annotation file formats, or not designed to deal with very dense annotation files such as repeated sequences. DensityMap can automatically compute the densities of features to give a series of windows along chromosomes—and this for a complete genome. It is very flexible; it can be used to analyse not just very dense annotations but also low density annotations by applying the computing and graphical options provided. It is also very efficient for plotting density maps of total repeats – satellites, TEs, simple sequence repeats - of human genome – 5 295 850 features – in 2 min 14 second a on computer equipped of a Intel(R) Xeon(R) W3670 CPU @ 3.20GHz and 16 Go of RAM. DensityMap is very simple to install and run, and so is a good way to obtain a global view of genomic data. To make easier the usage of DensityMap to persons non initiate to linux command line, we developed a web graphical user interface for online DensityMap analysis.

### Availability and requirements

- Project name: DensityMap.pl
- Project home page: https://github.com/sguizard/DensityMap
- Graphical user interface: http://chicken-repeats.inra.fr/launchDM_form.php
- Operating system(s): Linux
- Programming language: Perl
- Other requirements: Perl module GD::SVG
- License: GNU GPL v3
- Restrictions on its non-academic use: None

## Additional files

Additional file 1: Synopsis of the main program and functions. (DOCX 6 kb) Additional file 2: DensityMap Manual. (DOCX 239 kb)

## Abbreviations

BED: browser extensible data
Bp: base pair
CNV: copy number variation
CPU: central processing unit
CSV: comma-separated values
DNA: deoxyribonucleic acid
GFF: generic feature format
LINE: long interspersed nuclear element
LTR: long terminal repeat
Mbp: mega base pair
RAM: random access memory
SNP: single nucleotide polymorphism
SVG: scalable vector graphics
TE: transposable element

## References

[1] Batley J, Edwards D (2009). Genome sequence data: Management, storage, and visualization. Biotechniques.

[2] Kent WJ, Sugnet CW, Furey TS, Roskin KM, Pringle TH (2002). The Human Genome Browser at UCSC. Genome Res.

[3] Wang J, Kong L, Gao G, Luo J (2013). A brief introduction to web-based genome browsers. Brief Bioinformatics.

[4] Lee E, Helt G, Reese JT, Munoz-Torres MC, Childers CP (2013). Web Apollo: a web-based genomic annotation editing platform. Genome Biol.

[5] Krzywinski M, Schein J, Birol I, Connors J, Gascoyne R (2009). Circos: An information aesthetic for comparative genomics. Genome Res.

[6] An J, Lai J, Sajjanhar A, Batra J, Wang C (2015). J-Circos: an interactive Circos plotter. Bioinformatics.

[7] Pont C, Murat F, Guizard S, Flores R, Foucrier S (2013). Wheat syntenome unveils new evidences of contrasted evolutionary plasticity between paleo- and neoduplicated subgenomes. Plant J.

[8] Wolfe D, Dudek S, Ritchie MD, Pendergrass S (2013). Visualizing genomic information across chromosomes with PhenoGram. BioData Mining.

[9] Cannon EKS, Cannon SB (2011). Chromosome visualization tool: a whole genome viewer. International J plant genom.

