# Supplementary material for: DensityMap: a genome viewer for illustrating the densities of features
Source: BMC Bioinformatics. 2016 May 6;17:204. doi: 10.1186/s12859-016-1055-0 (PMC4858867; doi:10.1186/s12859-016-1055-0)
Supplement: Additional file 1: — Synopsis of the main program and functions. (DOCX 6 kb) [file 12859_2016_1055_MOESM1_ESM.docx]

1. Main
2. Check mandatory options
3. Read optional options and set default values if needed
4. Image size computation
   1. Height computation
      1. For each GFF files
         1. Increment number of chromosome to plot
         2. Check the first line for gff version
         3. Check the second line for chromosome length
         4. Store the highest chromosome length
      2. if auto_scale_factor
         1. while picture height > max picture height
            1. compute picture height
            2. end loop if condition is satisfied
            3. multiply scale factor by 10
      3. Store picture height
   2. Width computation
      1. For each couple of type=value
         1. define number of strand to plot
         2. store total number of strand to plot
      2. Compute and store picture width
5. Ask user if picture dimensions are fine
6. Create pictures
7. Load colours and colours schemes from colours.txt
8. if background is set draw background
9. if title add title to the picture
10. if show_scale add scale to the picture (drawScale)
11. Prepare data structure to store intervals for each strands
12. Draw strands of all chromosomes: For each GFF
    1. Open GFF file and read chromosome length
    2. Clean data structure of previous intervals
    3. For each chromosome of the GFF file
       1. skip line if current line type is not in user's type list
       2. if current line type = centromere, Store centromere positions
       3. store intervals in data structure
13. if label_strand_rotation Apply rotation to labels
14. Remove stroke of each window
15. Save picture
16. ProcessData
17. For each type
    1. for each strand
       1. sort intervals
18. For each type
    1. for each strand
       1. Reduce intervals (removeIntervalRedundancy)
19. For each type
    1. for each strand
       1. set position of first window in picture
20. For each type
    1. for each strand
       1. draw all windows in picture (drawPixels)
21. removeIntervalRedundancy
22. For each intervals collection
    1. Load first interval (Once)
    2. if current interval start < previous interval end
       1. replace previous interval end by current interval end
    3. else if current interval end < previous interval end
       1. remove interval
    4. else (current interval start > previous interval end)
       1. set current intervals as previous interval
23. Return reduce intervals collection
24. drawScale
25. Search the max number of scale ticks, start with 10 bases by ticks
    1. while number of scale ticks > max number of scale ticks
       1. compute number of ticks
       2. end loop if condition is satisfied
       3. multiply number of base by tick by 10
26. Add scale unit to picture
27. Add number of base by window to picture
28. Draw scale to picture
29. Draw last tick to picture
30. Draw other ticks to picture
31. drawPixels
32. Get unique id for svg strand group
33. open svg strand group
34. For each window
    1. load number of bases covered by previous interval (interval spanning several window(s), interval size > window size)
    2. while end of interval collection is not reached and interval is in current window
       1. if interval is in current window
          1. increment number of covered bases by interval size
       2. if interval is across current and next window (and more)
          1. compute number of windows covered by the intervals
          2. increment number of covered bases for the current window
          3. For each windows fully covered by interval
             1. store covered bases for each window
          4. Store the last window number of bases covered by the interval
    3. Compute percentage of window covered bases (=density)
    4. Draw window to the picture with colour corresponding to the density
35. Draw centromere to picture
36. Draw sequence name label
37. Draw type name label
38. Draw strand name label
39. Close strand group
