# Supplementary material for: DensityMap: a genome viewer for illustrating the densities of features
Source: BMC Bioinformatics. 2016 May 6;17:204. doi: 10.1186/s12859-016-1055-0 (PMC4858867; doi:10.1186/s12859-016-1055-0)
Supplement: Additional file 2: — DensityMap Manual. (DOCX 239 kb) [file 12859_2016_1055_MOESM2_ESM.docx]

DensityMap

Table of content

[1 Purpose 2](#__RefHeading___Toc105_1947720891)

[2 Installation 2](#__RefHeading___Toc107_1947720891)

[2.1 Requirements 2](#__RefHeading___Toc109_1947720891)

[2.2 DensityMap Installation 2](#__RefHeading___Toc111_1947720891)

[3 Directions for use 3](#__RefHeading___Toc113_1947720891)

1. Purpose

The visualization of genomic data is a considerable challenge. Many tools, such as Gbrowse, Jbrowse, and Abrowse, are available for displaying the data for small genomic loci, but few have attempted to visualize whole chromosomes or genomes. Phenogram and CviT offer two solutions to this problem but each has its own limitations. We needed a program suitable for treating repeated sequences, which can be very numerous in some genomes (45 % in the human genome, over 90 % in the wheat genome). We therefore designed DensityMap, which can represent the density (number of base pairs covered) of one or more features along chromosomes using a series of windows. The features correspond to the third column of the GFF annotation files (format: <http://www.sequenceontology.org/gff3.shtml>) used as inputs. The program generates high quality SVG pictures that can be edited.

This program makes it possible to represent the contents of GFF files simply. It allows the user to select the data to be plotted, provides automatic chromosome scaling, and an output picture that is easily configured using the many graphics options provided.

1. Installation
   1. Requirements

DensityMap is in Perl script that needs only one requirement to work, GD::SVG (tested with version 0.33-1). GD::SVG libraries can be installed on a Debian-based system using the apt-get package manager:

sudo apt-get install libgd-svg-perl

It also use POSIX libraries that are included in Linux distributions for ceiling and floor functions. Term::ANSIColor libraries can be installed as options for more colourful output. Download the Perl module from cpan:

<https://metacpan.org/pod/Term::ANSIColor>.

Then install it following the standard Perl module installation procedure:

perl Makefile.PL

make

make test

sudo make install

Activate the colour terminal output by uncommenting lines 16 and 17 in DensityMap.pl script.

2.2 DensityMap Installation

Download the DensityMap archive or clone repository from github:

<https://github.com/sguizard/DensityMap>.

The program can be launched directly from the unzipped archive or you can execute the install script for an all-users installation:

sudo install.sh

It will create a directory DensityMap in /usr/local/share and copy all files in it. It then updates the path of colours.txt in DensityMap.pl and creates a symbolic link to /usr/local/bin.

1. Use

Mandatory options

- -i, --input
  - string
  - Name of Gff file(s)
- -re, --region_file
  - string
  - Name of a BED file describing sequences regions to plot.
- -o , --output_img_name
  - string
  - Name of the output image, this extension .svg will be automatically added
- -ty, --type_to_draw
  - string
  - List of type to draw, strand to plot and colour scale to use
  - Format: "Type1=strand;Type2=strand=8"
    - Type (third column of GFF): match, gene, CDS, etc.
    - Strand:
      - - → strand -
      - + → strand +
      - both → strand - and strand +
      - fused → Combination of strand - and strand +
      - all → strand - and strand + and fused

Generic options

- -v, --verbose: more text explanation
- -h, --help: This help
- -for, --force: Automatically answers yes to picture size validation

Density options

- -c, --colour_scale:
  - integer
  - colour scale to use
  - (Default = 7)
- -sc, --scale_factor:
  - integer
  - window length in bp
  - (Default = 1000)
- -a, --auto_scale_factor:
  - integer
  - Max picture height in pixel
- -ro, --rounding_method:
  - string
  - floor or ceil
  - (Default = floor)
- -gc, --gc:
  - integer
  - colour scale to use
  - Create a density map of the GC% along the chromosome, REQUIRE the presence of the fasta sequence in the ##FASTA section of the GFF file

Graphical options

- -ti, --title
  - string
  - Title to print on the picture
- -w, --win_size
  - integer
  - Height of window in pixel
  - Default: 1
- -sh, --show_scale
  - integer
  - Draw Scale, n = num max ticks
  - Default: 50
- -str_w, --str_width
  - integer
  - Strand width in pixel
  - Default: 50
- -str_s, --str_space
  - integer
  - Space between strands in pixels
  - Default: 50
- -sp, --space_chr
  - integer
  - space between chromosomes in pixels
  - default: 50
- -lm, --lmargin
  - integer
  - left margin in pixels
  - default: 50
- -rm, --rmargin
  - integer
  - right margin in pixels
  - default: 50
- -tm, --tmargin
  - integer
  - top margin in pixels
  - default: 50
- -bm, --bmargin
  - integer
  - bottom margin in pixels
  - default: 50
- -ba, --background
  - color
  - fill background
  - default: no background
- -la, --label_strand_rotation
  - integer
  - rotation degree of strand label
  - default: 0
- -ft_f, --ft_family:
  - string
  - font to use for text
  - default: “Helvetica”
- -ft_s, --ft_size:
  - integer
  - font size
  - default: 16

Running the program:

The GFF set as the input will only be valid if it respects the format defined in: <http://www.sequenceontology.org/gff3.shtml>. It also must contain the header sequence-region for each chromosome.

Example of valid file:

##sequence-region 2L 1 23513712

2L RefSeq gene 7529 9484 . + . ID=gene2671

2L RefSeq gene 9839 21376 . - . ID=gene2672

…

2L RM LTR 23512506 23512653 809 + . Target=DM297_I-int 3285 3432

##sequence-region 2R 1 25286936

2R RefSeq gene 432219 705848 . + . ID=gene6156

…

##FASTA

>seq1

ALONGSEQ...

The mandatory options must be set to create a basic image of your data:

DensityMap.pl -i 2R.gff3 -ty "LTR=fused" -o 2R

This program is also executed with default graphical options (see above list) and will compute the size of the output picture and ask you if you want to continue or stop the execution. As you can see, the picture produced will be over 25,000 pixels high.

Two options are available for producing a reasonable picture height. You can define the window size to use to compute the density of feature using the -sc option. Or you can let the program choose for you and define a maximum picture height with the option -a.

You can try using the same command with the option -a 3000 to obtain a picture whose height does not exceed 3000 pixels and add the -ba white option to obtain a white background.

DensityMap.pl -i 2R.gff3 -ty "LTR=fused" -o 2R -a 3000 -ba white

The program will ask for the window size (scale factor) and ask you to validate the picture height. The resulting picture is 150 px by 2653 px, which is easily viewed (Figure 1). The default colour scale is blue to red: nucleotide windows with low-density features (LTR) to nucleotide windows with high-density features.

Several chromosomes can be visualized by pooling the GFF files on the -g option. Information for different types can also be displayed by defining them in the -ty option. The graphical options you can be used to add a scale and a title to the picture (Figure 2).

DensityMap.pl -i dmel.gff3 -o dmel -ty "LTR=fused;LINE=fused" -ba white -sc 20000 -sh 100 -title "LTR and LINE retrotransposon in Dmel genome"

This gives you a density map of the whole genome of *Drosophilia Melanogaster* describing the density of LINE and LTR retrotransposons. As you can see, the density of LTR retrotransposons is higher than that of LINE, but both TEs dispjay a similar distribution with high concentrations at the ends of each chromosome and large areas devoid of transposable elements, except for chromosomes 4 and X which are almost devoid of transposable elements.

DensityMap can be also used to study low density features like rolling circle transposons. You can use another colour scale, like number 9. It is designed to set a blank pixel for windows with a density of 0 - 1 % and a red pixel for windows with a density over 1 %. You also modify the way the density is rounded. Densities are rounded down using floor method by default, so a density of < 1 % is rounded to 0 %. Setting the rounding method (-ro) to ceiling rounds the value up to 1 %. You should use the ceil method if you need to maximize the visibility of low density features (Figure 3).

DensityMap.pl -i dmel.gff3 -o RC -ty "RC=fused" -ba white -sc 20000 -sh 100 -title "Rolling Circle transposons in Dmel genome" -c 9 -ro ceil

If you know the positions of the centromeres on the chromosomes you can add their coordinates to the GFF file with the type centromere. They will then be displayed on chromosomes as shown in Figure 3 for chromosome 3R.

You can modify all the picture margins (bottom, top, left, right), the space between strands and the width of the strands.

Customizing the colour scheme:

DensityMap is delivered with 10 colour scales for representing densities. If you find no suitable colour scale for your representation, you can create your own by modifying the colours.txt installation directory. A colour scale is composed of 101 colours, and each contains four pieces of information:

- An id formatted: <ID>_heatmap<COLOURNUMBER>
- A Red value, 0 to 255
- A Green value, 0 to 255
- A Blue value, 0 to 255

These specifications are separated by semi-colons.

Example of colour scale:

1_heatmap10;0;200;0

1_heatmap11;0;195;0

…

1_heatmap99;245;0;0

1_heatmap100;250;0;0

The DensityMap archive contains a script that reads the colours.txt file and creates a miniature of all the colour scales available (Figure 4). You only need to execute the scaleColorDrawer.pl script in the colours.txt directory without any arguments.


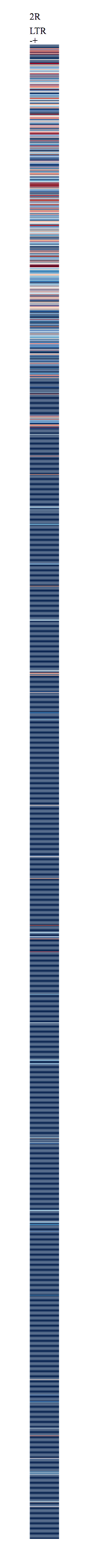
Figure 1


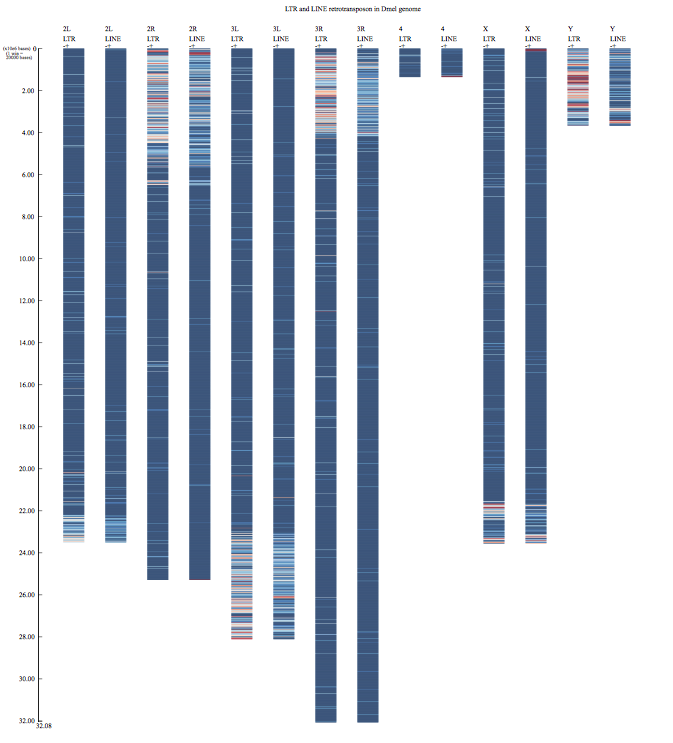
Figure 2


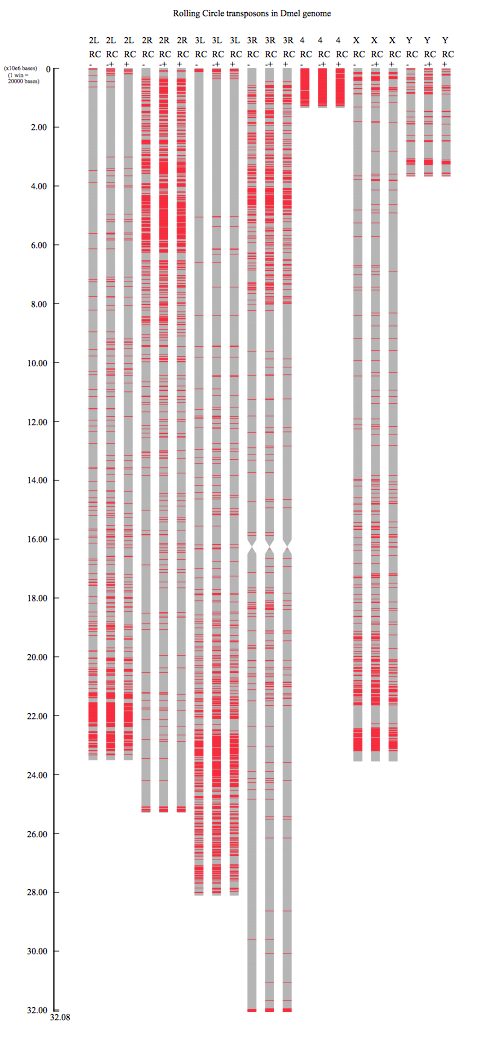
Figure 3


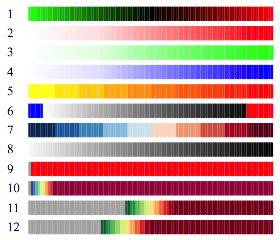
Figure 4:
